# Cervical spine tuberculosis

**DOI:** 10.11604/pamj.2020.37.7.25226

**Published:** 2020-09-02

**Authors:** Maher Teka, Hazem Ben Ghozlen, Akram Yassine Zaier, Majdi Ben Hnia, Nader Naouar, Faouzi Abid

**Affiliations:** 1 Department of Orthopaedics and Trauma, Taher Sfar Hospital of Mahdia, Mahdia, Tunisia,; 2 Department of Orthopaedics and Trauma, Sahloul Hospital of Sousse, Sousse, Tunisia

**Keywords:** Arthrodesis, tuberculosis, spinal tuberculosis, spinal arthrodesis

## Abstract

Tuberculosis of the cervical spine differs from other vertebral localizations by its extreme rarity, the clinical images are very diversified, the radiological measurements allow a good diagnostic orientation and specifically the MRI which allows a multi-planar study of the various lesions. Only bacteriological evidence can confirm the diagnosis. The treatment is based on a 12-month antituberculosis multidrug therapy and much debate upon the surgical indication. In our case, the patient presented with bilateral cervicobrachialgia with pain on examination at the mobilization of the cervical spine. A standard X-ray, a cervical CT scan, and a cervical MRI were performed, showing a C4 vertebral body compression of a probably infectious origin. The biopsy confirmed the diagnosis of a Cervical Pott's Disease that had been treated with anterior arthrodesis and TB treatment with rehabilitation, the patients' neurological symptoms improved, and he was doing well.

## Introduction

The most common extra-pulmonary skeletal form of tuberculosis is within the spine, with a predilection for the thoracic and lumbar regions [1]. Tuberculosis of the cervical spine is distinguished from other vertebral localizations by its extreme rarity, representing approximately 2-3% of spinal TB cases [1-3]. Therefore, its clinical and radiological semiology is quite distinctive, as the cervical spine can be affected by lesions that lead to instability and neurological deficits, and its prognosis is conditioned by the risk of bulbo-medullary compression. The conduct of the cervical spine TB can involve a variety of interventions, ranging from TB treatment to surgery, although chemotherapy is the mainstay of the treatment [2].

## Patient and observation

**Figure 1:**
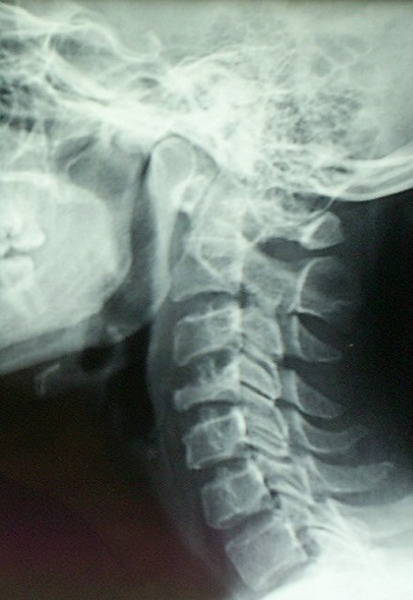
standard radiography showed osteolysis with compression of the vertebral body of C4

**Figure 2:**
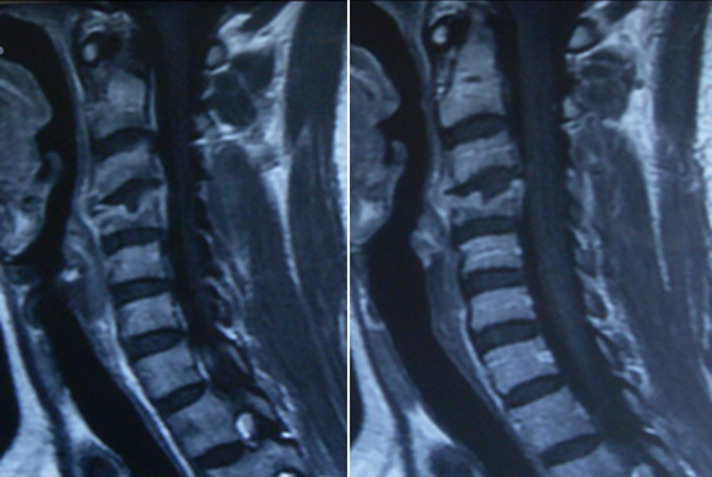
magnetic resonance imaging revealed a compression of the C4 body with epiduritis, or spinal epidural abscess, and infiltration of the paravertebral soft tissues, suggesting primarily an infectious origin

**Figure 3:**
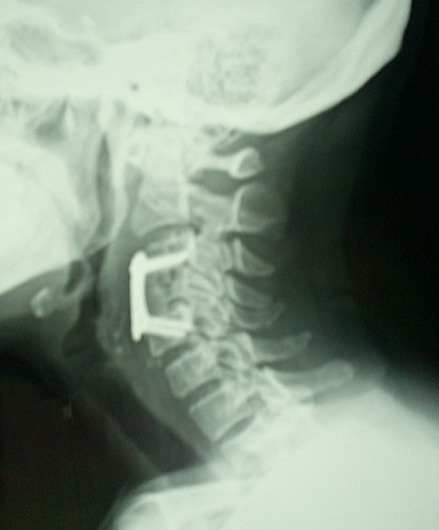
anterior arthrodesis

Patient K.R., 42 years old, without any particular pathological antecedents, who has had bilateral cervicobrachialgia for the past 9 months without any sensorimotor deficit, evolving in an unquantified weight loss context. On clinical examination, there was noticeable pain on the mobilization of the cervical spine. Standard radiography showed osteolysis with compression of the body of C4 (Figure 1). Magnetic resonance imaging confirmed the CT scan data and revealed a compression of the C4 vertebral body with epiduritis, or spinal epidural abscess and infiltration of the paravertebral soft tissues, suggesting primarily an infectious origin (Figure 2). The biopsy confirmed the diagnosis of active tuberculosis. The patient was treated with an anterior arthrodesis (Figure 3) and quadruple tuberculosis chemotherapy for 12 months. Post-rehabilitation showed improvement in patients' neurological symptoms, and his wellbeing with no signs of tuberculosis recurrence.

## Discussion

The tuberculosis localization in the cervical spine is very rare [3]. The clinical pictures are varied and can range from isolated neuralgia cervicobrachial to tetraplegia [4]. Weight loss, night fever, cervical lymphadenopathies and retropharyngeal abscesses should be brought to the attention of the clinician [4,5]. Radiological data is propitious for diagnosis, but CT and MRI scans allow a better definition of bone and soft tissue abnormalities. for a precise multi-planar study MRI is more accurate than a CT scan [3,6,7], it achieves a better tissue differentiation, allows a demonstration of the marrow in relation to the envelopes, the study of the spinal disc, and the tissue surrounding of the spine. It can also detect the earliest signs of disco-vertebral tubercular disease at an infra-radiological stage on both regular X-rays and CT scans [3,6,7]. Only bacteriological proof, either by biopsy of pre-vertebral soft tissues or by direct access to the focal point, can confirm the diagnosis [7,8], but sometimes the presumptive diagnosis is based on epidemiological, clinical, and radiological evidence. The treatment is based on poly anti-tuberculosis chemotherapy lasting approximately 12 months. The surgical indications are still much controversial [7,9]. The persistence of bone destruction with spinal instability is an indicator of surgical intervention. This treatment must be urgent in given the aggravation of a probable spinal cord compression [7]. Healing results in weight gain, biological normalization, and bone reconstruction with the fusion of bone lysed structures on X-ray.

## Conclusion

The cervical localization of Pott's disease is extremely rare. The clinical and radiological findings are often deceptive, requiring the use of modern imaging equipment to enable an accurate diagnosis of anatomical lesions. Treatment is based on antibiotic therapy and immobilization of the cervical spine.
